# Supplementary material for: The interactome of intact mitochondria by cross-linking mass spectrometry provides evidence for coexisting respiratory supercomplexes
Source: Mol Cell Proteomics. 2017 Dec 8;17(2):216–32. doi: 10.1074/mcp.RA117.000470 (PMC5795388; doi:10.1074/mcp.RA117.000470)
Supplement: Supplemental Data [file supp_RA117.000470_133922_0_supp_23978_fzffwf.zip › spectra_annotation/mito_spectra_annotation/234-1-5-1-7-1.pdf]

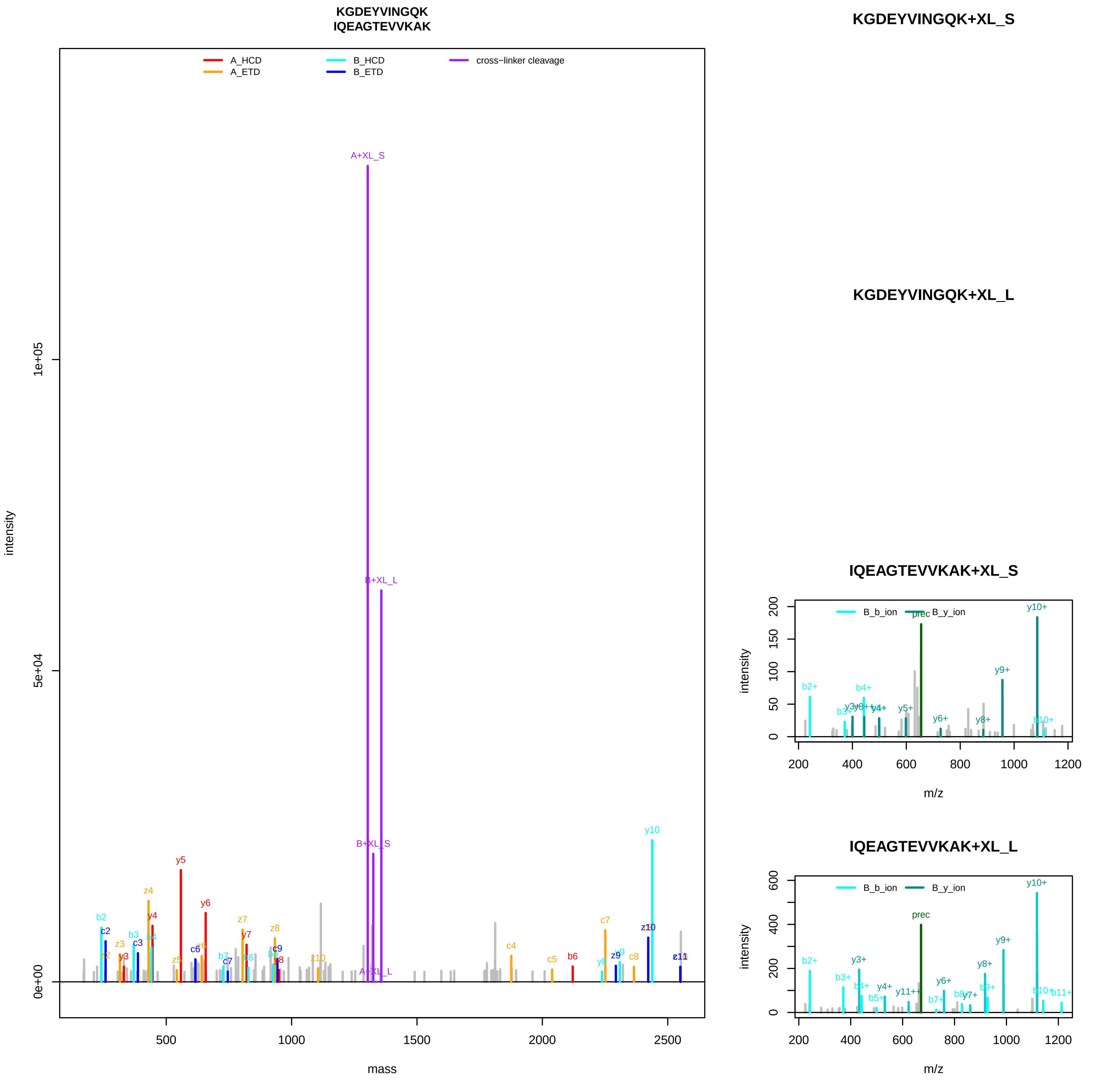

KGDEYVINGQK
IQEAGTEVVKAK
A_HCD
A_ETD
B_HCD
B_ETD
cross−linker cleavage
A+XL_S
B+XL_L
B+XL_S
A+XL_L
y10
y5
y6
y4
y7
z4
z7
z8
c4
c7
c5
b6
c8
z10
c2
c3
c6
c7
c9
c11
b2
b3
b7
intensity
mass
1e+05
5e+04
0e+00
500
1000
1500
2000
2500
KGDEYVINGQK+XL_S
KGDEYVINGQK+XL_L
IQEAGTEVVKAK+XL_S
IQEAGTEVVKAK+XL_L
B_b_ion
B_y_ion
prec
y10+
y9+
y8+
y6+
y5+
b2+
b3+
b4+
b10+
b11+
y3+
y4+
y7+
y11++
b5+
b7+
m/z
200
400
600
800
1000
1200
0
50
100
150
200
400
600
